# Supplementary material for: SARS-CoV-2 rapid antibody test results and subsequent risk of hospitalisation and death in 361,801 people
Source: Nat Commun. 2023 Aug 16;14:4957. doi: 10.1038/s41467-023-40643-w (PMC10432566; doi:10.1038/s41467-023-40643-w)
Supplement: Supplementary file 1 — Supplementary Information [file 41467_2023_40643_MOESM1_ESM.pdf]

Supplementary material

SARS-CoV-2 rapid antibody test results and subsequent risk of hospitalisation and death in 361,801 people

Matthew Whitaker, Bethan Davies, Christina Atchison, Wendy Barclay, Deborah Ashby, Ara Darzi, Steven Riley, Graham Cooke, Christl A. Donnelly, Marc Chadeau-Hyam, Paul Elliott, Helen Ward

---

Supplementary Tables 1-5

Supplementary Figures 1-3

Supplementary Methods

Supplementary Table 1 Hazard ratios from multiple Cox regression models with each outcome as the dependent variable, and varying degrees of adjustment (risk group, vaccination status, age, age^2, sex, prior COVID-19 status) among the full cohort. The independent variable of interest is the LFIA test result (time to event). 95% confidence intervals shown in square brackets. Asterisks indicate statistical significance (see table footnote). P-values are derived from a two-sided t-test on the coefficient with the null hypothesis that the true value of the coefficient is zero. P-values are not adjusted for multiple comparison.

Predictor	N	N events	Crude	Plus at risk	Plus vaccination status	Plus age and age^2	Plus sex	Prior COVID-19
**Hospitalisation**								
		16802.0	1.15 [1.11,1.19]****	1.24 [1.20,1.29]****	1.00 [0.95,1.04]	0.94 [0.90,0.98]**	0.94 [0.90,0.99]*	0.97 [0.93,1.02]
LFIA negative	361801	0						
High-risk group	361801	16802	-	3.04 [2.95,3.14]****	3.11 [3.01,3.21]****	2.58 [2.50,2.67]****	2.59 [2.51,2.68]****	2.59 [2.50,2.68]****
1 vaccine	361801	16802	-	-	0.62 [0.59,0.65]****	0.53 [0.50,0.55]****	0.53 [0.50,0.55]****	0.54 [0.51,0.56]****
2 vaccines	361801	16802	-	-	0.70 [0.66,0.74]****	0.48 [0.46,0.51]****	0.48 [0.46,0.51]****	0.50 [0.47,0.53]****
Age	361801	16802	-	-	-	0.99 [0.98,0.99]****	0.99 [0.98,0.99]****	0.99 [0.98,0.99]****
Age^2	361801	16802	-	-	-	1.00 [1.00,1.00]****	1.00 [1.00,1.00]****	1.00 [1.00,1.00]****
Male sex	361801	16802	-	-	-	-	0.91 [0.89,0.94]****	0.91 [0.89,0.94]****
Prior COVID-19	361801	16802	-	-	-	-	-	1.11 [1.07,1.16]****
**Emergency hospitalisation**								
LFIA negative	361801	5330	1.23 [1.16,1.31]****	1.34 [1.26,1.43]****	0.93 [0.86,1.01]	0.89 [0.82,0.96]**	0.89 [0.82,0.96]**	0.96 [0.89,1.05]
High-risk group	361801	5330	-	3.28 [3.10,3.47]****	3.43 [3.24,3.64]****	2.94 [2.77,3.12]****	2.95 [2.78,3.13]****	2.93 [2.76,3.11]****
1 vaccine	361801	5330	-	-	0.53 [0.49,0.58]****	0.49 [0.45,0.53]****	0.49 [0.45,0.53]****	0.51 [0.47,0.56]****
2 vaccines	361801	5330	-	-	0.52 [0.47,0.58]****	0.39 [0.35,0.43]****	0.39 [0.35,0.43]****	0.42 [0.38,0.47]****
Age	361801	5330	-	-	-	0.95 [0.94,0.96]****	0.95 [0.94,0.96]****	0.95 [0.94,0.96]****
Age^2	361801	5330	-	-	-	1.00 [1.00,1.00]****	1.00 [1.00,1.00]****	1.00 [1.00,1.00]****
Male sex	361801	5330	-	-	-	-	0.94 [0.89,1.00]*	0.94 [0.89,1.00]*

Predictor	N	N events	Crude	Plus at risk	Plus vaccination status	Plus age and age²	Plus sex	Prior COVID-19
Prior COVID-19	361801	5330	-	-	-	-	-	1.28 [1.19,1.38]****
**Hospitalisation with COVID-19 primary diagnosis**								
LFIA negative	361801	91	1.62 [0.97,2.70]	1.74 [1.04,2.90]*	1.50 [0.76,2.95]	1.46 [0.74,2.89]	1.42 [0.72,2.81]	1.92 [0.99,3.71]
High-risk group	361801	91	-	2.86 [1.84,4.46]****	2.81 [1.79,4.40]****	2.53 [1.60,4.00]****	2.48 [1.57,3.93]***	2.41 [1.53,3.82]***
1 vaccine	361801	91	-	-	0.32 [0.14,0.74]**	0.25 [0.11,0.59]**	0.25 [0.11,0.59]**	0.29 [0.13,0.67]**
2 vaccines	361801	91	-	-	0.93 [0.44,1.98]	0.80 [0.37,1.76]	0.81 [0.37,1.77]	1.04 [0.49,2.23]
Age	361801	91	-	-	-	1.19 [1.06,1.34]**	1.19 [1.06,1.34]**	1.18 [1.05,1.33]**
Age²	361801	91	-	-	-	1.00 [1.00,1.00]**	1.00 [1.00,1.00]**	1.00 [1.00,1.00]*
Male sex	361801	91	-	-	-	-	1.56 [1.03,2.36]*	1.55 [1.02,2.36]*
Prior COVID-19	361801	91	-	-	-	-	-	2.62 [1.64,4.18]****
Death								
LFIA negative	361801	389	1.69 [1.31,2.17]****	1.99 [1.54,2.57]****	1.88 [1.37,2.57]***	1.55 [1.12,2.15]**	1.52 [1.10,2.10]*	1.53 [1.10,2.13]*
Higher-risk group	361801	389	-	8.44 [6.90,10.32]****	8.41 [6.86,10.32]****	4.61 [3.73,5.68]****	4.47 [3.62,5.52]****	4.47 [3.62,5.51]****
1 vaccine	361801	389	-	-	0.71 [0.51,0.98]*	0.46 [0.34,0.63]****	0.46 [0.34,0.63]****	0.46 [0.34,0.63]****
2 vaccines	361801	389	-	-	0.95 [0.66,1.35]	0.41 [0.29,0.59]****	0.41 [0.28,0.59]****	0.41 [0.28,0.59]****
Age	361801	389	-	-	-	0.95 [0.91,0.99]*	0.94 [0.90,0.99]*	0.94 [0.90,0.99]*
Age²	361801	389	-	-	-	1.00 [1.00,1.00]****	1.00 [1.00,1.00]****	1.00 [1.00,1.00]****
Male sex	361801	389	-	-	-	-	1.53 [1.25,1.88]****	1.53 [1.25,1.88]****
Prior COVID-19	361801	389	-	-	-	-	-	1.04 [0.75,1.45]
**Death with COVID-19 underlying†**								
LFIA negative	361801	20	7.06 [0.92,54.10]	8.50 [1.10,65.48]*	12.30 [1.32,114.44]*	11.62 [1.23,109.39]*	11.29 [1.20,106.20]*	12.88 [1.40,118.56]*
Higher-risk group	361801	20	-	11.29 [4.44,28.70]****	10.58 [4.11,27.27]****	6.59 [2.53,17.20]***	6.39 [2.44,16.73]***	6.22 [2.37,16.29]***
1 vaccine	361801	20	-	-	0.39 [0.05,3.13]	0.30 [0.04,2.35]	0.29 [0.04,2.33]	0.31 [0.04,2.47]

Predictor	N	N events	Crude	Plus at risk	Plus vaccination status	Plus age and age²	Plus sex	Prior COVID-19
2 vaccines	361801	20	-	-	2.13 [0.47,9.59]	1.26 [0.28,5.73]	1.25 [0.28,5.64]	1.35 [0.30,6.06]
Age	361801	20	-	-	-	1.23 [0.85,1.78]	1.22 [0.84,1.78]	1.23 [0.85,1.79]
Age²	361801	20	-	-	-	1.00 [1.00,1.00]	1.00 [1.00,1.00]	1.00 [1.00,1.00]
Male sex	361801	20	-	-	-	-	1.64 [0.64,4.18]	1.66 [0.65,4.23]
Prior COVID-19	361801	20	-	-	-	-	-	2.46 [0.80,7.62]
**Death with COVID-19 mentioned†**								
LFIA negative	361801	20	7.85 [1.03,59.66]*	9.41 [1.23,71.89]*	12.54 [1.36,115.74]*	11.81 [1.26,110.40]*	11.39 [1.22,106.23]*	12.77 [1.40,116.87]*
Higher-risk group	361801	20	-	10.72 [4.44,25.88]****	10.18 [4.17,24.88]****	6.23 [2.52,15.42]****	5.98 [2.41,14.86]***	5.85 [2.36,14.55]***
1 vaccine	361801	20	-	-	0.34 [0.04,2.68]	0.26 [0.03,2.01]	0.25 [0.03,1.99]	0.27 [0.03,2.08]
2 vaccines	361801	20	-	-	1.86 [0.43,8.12]	1.09 [0.25,4.79]	1.07 [0.24,4.70]	1.14 [0.26,4.97]
Age	361801	20	-	-	-	1.23 [0.86,1.76]	1.23 [0.86,1.75]	1.23 [0.86,1.76]
Age²	361801	20	-	-	-	1.00 [1.00,1.00]	1.00 [1.00,1.00]	1.00 [1.00,1.00]
Male sex	361801	20	-	-	-	-	1.92 [0.77,4.78]	1.94 [0.78,4.82]
Prior COVID-19	361801	20	-	-	-	-	-	2.16 [0.71,6.59]

**p<0.05, **p<0.01, ***p<0.001, **** p<0.000*

†small number suppression applied

Supplementary Table 2 Hazard ratios from multiple Cox regression models with each outcome as the dependent variables, and varying degrees of adjustment (risk group, vaccination status, age, age², sex, prior COVID-19 status), among N=192,604 participants who had received one or more vaccinations at least 14 days before taking the LFIA test. The independent variable of interest is the LFIA test result. 95% confidence intervals shown in square brackets. Asterisks indicate statistical significance (see table footnote). P-values are derived from a two-sided t-test on the coefficient with the null hypothesis that the true value of the coefficient is zero. P-values are not adjusted for multiple comparison.

Predictor	N	N events	Crude	Plus risk group	Plus doubly vaccinated	Plus age and age²	Plus sex	Plus prior COVID-19
Hospitalisation								
LFIA negative	192604	5615	0.96 [0.91,1.02]	0.99 [0.94,1.05]	1.08 [1.02,1.15]*	1.02 [0.97,1.09]	1.03 [0.97,1.09]	1.03 [0.97,1.09]
High-risk group	192604	5615	-	2.62 [2.48,2.77]****	2.50 [2.36,2.64]****	2.36 [2.23,2.49]****	2.32 [2.20,2.46]****	2.33 [2.20,2.46]****
2 vaccines	192604	5615	-	-	1.29 [1.21,1.36]****	1.06 [1.00,1.13]	1.03 [0.97,1.10]	1.03 [0.97,1.10]
Age	192604	5615	-	-	-	1.02 [1.02,1.03]****	0.95 [0.94,0.97]****	0.95 [0.94,0.97]****
Age²	192604	5615	-	-	-	-	1.00 [1.00,1.00]****	1.00 [1.00,1.00]****
Male sex	192604	5615	-	-	-	-	-	0.96 [0.91,1.01]
Prior COVID-19	192604	5615	-	-	-	-	-	-
Emergency hospitalisation								
LFIA negative	192604	1569	1.00 [0.90,1.11]	1.03 [0.93,1.15]	1.08 [0.97,1.21]	1.02 [0.91,1.14]	1.03 [0.92,1.15]	1.03 [0.92,1.15]
High-risk group	192604	1569	-	3.11 [2.81,3.44]****	3.03 [2.74,3.36]****	2.85 [2.57,3.16]****	2.74 [2.47,3.04]****	2.74 [2.47,3.04]****
2 vaccines	192604	1569	-	-	1.14 [1.02,1.28]*	0.95 [0.84,1.06]	0.90 [0.80,1.01]	0.90 [0.80,1.01]
Age	192604	1569	-	-	-	1.02 [1.02,1.03]****	0.88 [0.86,0.90]****	0.88 [0.86,0.90]****
Age²	192604	1569	-	-	-	-	1.00 [1.00,1.00]****	1.00 [1.00,1.00]****
Male sex	192604	1569	-	-	-	-	-	0.99 [0.89,1.09]
Prior COVID-19	192604	1569	-	-	-	-	-	-
Hospitalisation with COVID-19 primary diagnosis								

Predictor	N	N events	Crude	Plus risk group	Plus doubly vaccinated	Plus age and age²	Plus sex	Plus prior COVID-19
LFIA negative	192604	24	1.69 [0.76,3.76]	1.81 [0.81,4.05]	2.69 [1.15,6.31]*	2.67 [1.13,6.27]*	2.47 [1.05,5.81]*	2.73 [1.15,6.48]*
High-risk group	192604	24	-	4.52 [2.03,10.08]***	3.55 [1.56,8.07]**	3.56 [1.57,8.11]**	3.27 [1.43,7.47]**	3.24 [1.42,7.40]**
2 vaccines	192604	24	-	-	3.26 [1.26,8.49]*	3.35 [1.20,9.38]*	3.52 [1.24,9.96]*	3.65 [1.30,10.27]*
Age	192604	24	-	-	-	1.12 [0.79,1.59]	1.11 [0.79,1.57]	1.11 [0.79,1.57]
Age²	192604	24	-	-	-	1.00 [1.00,1.00]	1.00 [1.00,1.00]	1.00 [1.00,1.00]
Male sex	192604	24	-	-	-	-	3.40 [1.34,8.65]*	3.43 [1.35,8.72]**
Prior COVID-19	192604	24	-	-	-	-	-	2.62 [0.96,7.21]
Hospitalisation with COVID-19 mentioned								
LFIA negative	192604	32	1.36 [0.67,2.75]	1.45 [0.72,2.94]	1.93 [0.91,4.10]	1.86 [0.88,3.97]	1.74 [0.82,3.72]	2.07 [0.97,4.44]
High-risk group	192604	32	-	5.02 [2.50,10.05]****	4.24 [2.08,8.64]****	4.10 [2.01,8.36]***	3.80 [1.85,7.79]***	3.72 [1.82,7.64]***
2 vaccines	192604	32	-	-	2.33 [1.04,5.21]*	1.99 [0.85,4.69]	2.04 [0.86,4.84]	2.21 [0.94,5.19]
Age	192604	32	-	-	-	1.04 [0.80,1.34]	1.02 [0.80,1.32]	1.03 [0.80,1.33]
Age²	192604	32	-	-	-	1.00 [1.00,1.00]	1.00 [1.00,1.00]	1.00 [1.00,1.00]
Male sex	192604	32	-	-	-	-	2.83 [1.30,6.17]**	2.87 [1.32,6.26]**
Prior COVID-19	192604	32	-	-	-	-	-	3.93 [1.77,8.72]***
Death†								
LFIA negative	192604	122	1.67 [1.17,2.38]**	1.72 [1.20,2.47]**	2.07 [1.42,3.03]***	1.62 [1.09,2.41]*	1.58 [1.06,2.35]*	1.59 [1.07,2.37]*
High-risk group	192604	122	-	8.52 [5.85,12.41]****	7.61 [5.18,11.18]****	5.01 [3.40,7.38]****	4.82 [3.26,7.11]****	4.81 [3.26,7.09]****
2 vaccines	192604	122	-	-	1.78 [1.18,2.68]**	0.91 [0.59,1.40]	0.90 [0.59,1.39]	0.91 [0.59,1.40]
Age	192604	122	-	-	-	1.00 [0.83,1.21]	0.99 [0.82,1.20]	0.99 [0.82,1.20]
Age²	192604	122	-	-	-	1.00 [1.00,1.00]	1.00 [1.00,1.00]	1.00 [1.00,1.00]
Male sex	192604	122	-	-	-	-	1.58 [1.09,2.28]*	1.58 [1.09,2.29]*
Prior COVID-19	192604	122	-	-	-	-	-	1.14 [0.59,2.20]

**p<0.05, **p<0.01, ***p<0.001, **** p<0.0001*

†Death with COVID-19 excluded because of insufficient data (N events <8)

Supplementary Table 3 Hazard ratios from multiple Cox regression models with each outcome as the dependent variable, and varying degrees of adjustment (risk group, vaccination status, age, age², sex, prior COVID-19 status), among N=88,027 participants who had received two or more vaccinations at least 14 days before taking the LFIA test. The independent variable of interest is the LFIA test result. 95% confidence intervals shown in square brackets. Asterisks indicate statistical significance (see table footnote). P-values are derived from a two-sided t-test on the coefficient with the null hypothesis that the true value of the coefficient is zero. P-values are not adjusted for multiple comparison.

Variable	N	N events	Crude	Plus risk group	Plus age and age^2	Plus sex	Plus prior COVID-19
Hospitalisation							
LFIA negative	88027	2936	1.18 [1.08,1.30]***	1.10 [1.00,1.21]	1.06 [0.96,1.17]	1.06 [0.97,1.17]	1.07 [0.97,1.17]
High-risk group	88027	2936	-	2.33 [2.17,2.51]****	2.29 [2.13,2.46]****	2.29 [2.12,2.46]****	2.29 [2.13,2.47]****
Age	88027	2936	-	-	1.02 [1.02,1.02]****	0.96 [0.94,0.98]***	0.96 [0.94,0.98]***
Age^2	88027	2936	-	-	-	1.00 [1.00,1.00]****	1.00 [1.00,1.00]****
Male sex	88027	2936	-	-	-	-	0.96 [0.90,1.04]
Prior COVID-19	88027	2936	-	-	-	-	-
Emergency hospitalisation							
LFIA negative	88027	780	1.33 [1.11,1.58]**	1.21 [1.01,1.44]*	1.16 [0.97,1.39]	1.18 [0.98,1.41]	1.17 [0.98,1.41]
High-risk group	88027	780	-	2.83 [2.45,3.25]****	2.77 [2.41,3.19]****	2.76 [2.39,3.18]****	2.74 [2.38,3.16]****
Age	88027	780	-	-	1.02 [1.01,1.03]****	0.88 [0.85,0.91]****	0.88 [0.85,0.91]****
Age^2	88027	780	-	-	-	1.00 [1.00,1.00]****	1.00 [1.00,1.00]****
Male sex	88027	780	-	-	-	-	1.04 [0.91,1.21]
Prior COVID-19	88027	780	-	-	-	-	-

Variable	N	N events	Crude	Plus risk group	Plus age and age²	Plus sex	Plus prior COVID-19
Hospitalisation with COVID-19 primary diagnosis							
LFIA negative	88027	17	3.01 [1.11,8.15]*	2.61 [0.96,7.08]	2.65 [0.97,7.22]	2.41 [0.89,6.58]	2.79 [1.01,7.72]*
High-risk group	88027	17	-	4.97 [1.83,13.48]**	4.94 [1.82,13.43]**	4.28 [1.57,11.66]**	4.27 [1.57,11.62]**
Age	88027	17	-	-	1.15 [0.74,1.80]	1.14 [0.74,1.76]	1.14 [0.73,1.75]
Age^2	88027	17	-	-	1.00 [1.00,1.00]	1.00 [1.00,1.00]	1.00 [1.00,1.00]
Male sex	88027	17	-	-	-	5.40 [1.53,19.03]**	5.53 [1.57,19.52]**
Prior COVID-19	88027	17	-	-	-	-	4.42 [1.52,12.91]**
Hospitalisation with COVID-19 mentioned							
LFIA negative	88027	21	2.84 [1.14,7.05]*	2.42 [0.97,6.02]	2.40 [0.96,5.99]	2.20 [0.88,5.50]	2.54 [1.01,6.41]*
High-risk group	88027	21	-	5.38 [2.16,13.37]***	5.35 [2.15,13.31]***	4.68 [1.88,11.68]***	4.62 [1.85,11.53]**
Age	88027	21	-	-	1.02 [0.75,1.38]	1.00 [0.74,1.35]	1.00 [0.74,1.36]
Age^2	88027	21	-	-	1.00 [1.00,1.00]	1.00 [1.00,1.00]	1.00 [1.00,1.00]
Male sex	88027	21	-	-	-	4.70 [1.56,14.14]**	4.82 [1.60,14.55]**
Prior COVID-19	88027	21	-	-	-	-	4.49 [1.70,11.86]**
Death†							
LFIA negative	88027	69	2.47 [1.48,4.12]***	2.09 [1.25,3.50]**	1.92 [1.15,3.22]*	1.87 [1.11,3.12]*	1.87 [1.11,3.13]*
High-risk group	88027	69	-	5.85 [3.52,9.73]****	5.04 [3.02,8.41]****	4.79 [2.86,8.00]****	4.78 [2.86,8.00]****
Age	88027	69	-	-	0.88 [0.73,1.05]	0.86 [0.72,1.03]	0.86 [0.72,1.03]
Age^2	88027	69	-	-	1.00 [1.00,1.00]**	1.00 [1.00,1.00]**	1.00 [1.00,1.00]**
Male sex	88027	69	-	-	-	1.68 [1.03,2.76]*	1.68 [1.03,2.76]*
Prior COVID-19	88027	69	-	-	-	-	1.05 [0.42,2.62]

**p<0.05, **p<0.01, ***p<0.001, **** p<0.0001*

†Death with COVID-19 excluded because of insufficient data (N events <8)

Supplementary Table 4 Predictors of a negative LFIA test. Logistic regression models were constructed with LFIA negative (yes/no) as the binary outcome variable, and biological and demographic variables as predictors. Three model specifications are reported: 1) crude model, 2) age and sex adjusted, 3) fully mutually adjusted.

**Variable**	**Category**	**Crude model OR (95% CI)**	**Age and sex adjusted aOR (95% CI)**	**Fully mutually adjusted Fully aOR (95% CI)**
Age group	35-44 [reference]	-	-	-
	18-24	1.033 (0.985,1.083)	1.039 (0.991,1.09)	0.564 (0.527,0.603)
	25-34	1.131 (1.091,1.174)	1.141 (1.1,1.183)	0.763 (0.724,0.804)
	45-54	0.738 (0.715,0.761)	0.73 (0.708,0.753)	0.907 (0.868,0.947)
	55-64	0.371 (0.362,0.381)	0.365 (0.355,0.375)	1.156 (1.112,1.202)
	65-74	0.202 (0.197,0.208)	0.196 (0.191,0.202)	1.245 (1.193,1.3)
	74+	0.33 (0.318,0.343)	0.32 (0.308,0.332)	1.591 (1.506,1.681)
Sex	Female [reference]	-	-	-
	Male	1.235 (1.219,1.252)	1.36 (1.341,1.379)	1.336 (1.31,1.363)
Adiposity	Normal weight [reference]	-	-	-
	Obese	1.006 (0.987,1.025)	0.989 (0.97,1.009)	1.124 (1.094,1.154)
	Overweight	0.97 (0.955,0.986)	0.998 (0.981,1.016)	1.049 (1.025,1.073)
	Underweight	1.197 (1.121,1.279)	1.164 (1.085,1.248)	1.127 (1.029,1.235)
Ethnicity	White [reference]	-	-	-
	Asian	0.779 (0.749,0.81)	0.546 (0.524,0.569)	0.673 (0.633,0.715)
	Black	0.646 (0.603,0.692)	0.508 (0.473,0.546)	0.596 (0.534,0.665)
	Mixed	1.325 (1.236,1.421)	0.854 (0.794,0.918)	0.855 (0.773,0.946)

	Other	0.748 (0.696,0.805)	0.629 (0.583,0.68)	0.802 (0.719,0.895)
Index of multiple deprivation (IMD) quintile	3 [reference]	-	-	-
	1 - most deprived	1.051 (1.023,1.079)	0.937 (0.911,0.963)	1.003 (0.964,1.045)
	2	1.011 (0.988,1.033)	0.951 (0.929,0.974)	1.011 (0.978,1.045)
	4	0.956 (0.937,0.974)	0.984 (0.964,1.004)	0.974 (0.947,1.002)
	5 - least deprived	0.951 (0.933,0.969)	0.984 (0.965,1.004)	0.96 (0.933,0.987)
Risk group	Lower-risk group [reference]	-	-	-
	Higher-risk group	0.652 (0.641,0.664)	0.845 (0.829,0.861)	1.085 (1.051,1.12)
Comorbidities	No comorbidities [reference]	-	-	-
	1 comorbidity	0.782 (0.77,0.794)	0.915 (0.9,0.93)	1.065 (1.04,1.091)
	2+ comorbidities	0.787 (0.773,0.802)	0.935 (0.917,0.954)	1.194 (1.155,1.234)
Region	South East [reference]	-	-	-
	East Midlands	0.983 (0.96,1.006)	0.985 (0.961,1.009)	0.971 (0.938,1.005)
	East of England	0.965 (0.943,0.987)	0.955 (0.933,0.978)	0.974 (0.943,1.007)
	London	0.722 (0.704,0.741)	0.615 (0.599,0.632)	0.755 (0.726,0.785)
	North East	0.944 (0.909,0.98)	0.936 (0.9,0.974)	0.885 (0.837,0.935)
	North West	0.841 (0.821,0.861)	0.83 (0.809,0.851)	0.86 (0.83,0.891)
	South West	1.096 (1.068,1.125)	1.136 (1.106,1.168)	1.073 (1.033,1.114)
	West Midlands	0.94 (0.916,0.965)	0.936 (0.911,0.962)	0.954 (0.918,0.991)
	Yorkshire and The Humber	0.953 (0.925,0.982)	0.965 (0.936,0.995)	0.954 (0.914,0.996)
Current smoker	Not current cigarette smoker [reference]	-	-	-
	Current cigarette smoker	1.694 (1.65,1.738)	1.409 (1.371,1.448)	1.428 (1.374,1.484)
	Prefer not to say	1.112 (1.038,1.191)	0.959 (0.893,1.031)	1.136 (1.002,1.288)

Number of vaccines	0 [reference]	-	-	-
	1	0.148 (0.145,0.151)	0.121 (0.118,0.124)	0.077 (0.075,0.08)
	2	0.025 (0.024,0.025)	0.017 (0.017,0.018)	0.009 (0.009,0.01)
Previous case of COVID	No known previous COVID-19 [reference]	-	-	-
	Yes, confirmed by doctor but not tested	0.547 (0.511,0.585)	0.405 (0.377,0.435)	0.209 (0.189,0.231)
	Yes, confirmed by positive test	0.11 (0.105,0.115)	0.068 (0.064,0.071)	0.022 (0.021,0.024)
	Yes, my own suspicions	0.898 (0.879,0.918)	0.631 (0.616,0.645)	0.278 (0.269,0.287)

Supplementary Table 5 Hazard ratios from multiple Cox regression models for each outcome, and adjustment on age, vaccination status, and an interaction effect between LFIA result (binary categorical with positive as the reference category) and number of vaccinations (categorical variable with 0 vaccines as the reference category). The independent variable of interest is death/hospitalisation/emergency hospitalisation/hospitalisation with COVID-19/hospitalisation with COVID-19 mentioned on the admission record. 95% confidence intervals are shown in square brackets. Specification:

Y [*Time to event*] ~ *Age* + *Vaccination* + *LFIA_result* + *Vaccination*LFIA_result*

A Full cohort

Variable	Category	Death	Hospitalised	Hospitalised emergency	Hospitalised COVID-19	Hospitalised COVID-19 mentioned
Age	[*continuous*]	1.11 [1.10,1.12]****	1.03 [1.03,1.03]****	1.02 [1.02,1.03]****	1.02 [1.01,1.04]**	1.02 [1.01,1.03]**
Vaccination	1 vaccine	0.33 [0.17,0.66]**	0.44 [0.41,0.48]****	0.38 [0.33,0.44]****	0.12 [0.02,0.61]*	0.12 [0.02,0.57]**
	2 vaccines	0.30 [0.17,0.55]****	0.47 [0.43,0.51]****	0.37 [0.32,0.42]****	0.42 [0.15,1.19]	0.50 [0.19,1.31]
LFIA result	LFIA negative	1.17 [0.70,1.97]	0.85 [0.80,0.91]****	0.77 [0.70,0.86]****	0.89 [0.38,2.05]	1.11 [0.51,2.41]
LFIA*Vaccination interaction	LFIA negative : 1 vaccine	1.44 [0.67,3.12]	1.17 [1.06,1.29]**	1.23 [1.03,1.46]*	2.93 [0.46,18.46]	2.35 [0.38,14.37]
	LFIA negative : 2 vaccines	1.80 [0.87,3.73]	1.31 [1.16,1.46]****	1.64 [1.33,2.01]****	3.80 [1.07,13.48]*	2.59 [0.78,8.58]

B Low-risk group

Variable	Category	Death	Hospitalised	Hospitalised emergency	Hospitalised COVID-19	Hospitalised COVID-19 mentioned
Age	[*continuous*]	1.10 [1.08,1.12]****	1.03 [1.02,1.03]****	1.02 [1.01,1.02]****	1.02 [1.00,1.04]*	1.01 [1.00,1.03]
Vaccination	1 vaccine	0.15 [0.04,0.52]**	0.46 [0.41,0.50]****	0.39 [0.33,0.46]****	0.19 [0.03,1.07]	0.18 [0.03,0.94]*
	2 vaccines	0.30 [0.12,0.72]**	0.46 [0.42,0.50]****	0.36 [0.31,0.43]****	0.28 [0.07,1.21]	0.28 [0.07,1.11]
LFIA result	LFIA negative	1.19 [0.56,2.56]	0.82 [0.76,0.88]****	0.74 [0.65,0.83]****	1.02 [0.37,2.85]	1.18 [0.47,2.93]
LFIA*Vaccination interaction	LFIA negative : 1 vaccine	2.95 [0.77,11.29]	1.19 [1.06,1.33]**	1.22 [0.99,1.51]	2.03 [0.28,14.76]	1.80 [0.26,12.34]
	LFIA negative : 2 vaccines	0.70 [0.17,2.94]	1.22 [1.04,1.42]*	1.40 [1.04,1.89]*	4.30 [0.70,26.39]	3.83 [0.66,22.08]

C High-risk group

Variable	Category	Death	Hospitalised	Hospitalised emergency	Hospitalised COVID-19†	Hospitalised COVID-19 mentioned†
Age	[*continuous*]	1.09 [1.08,1.11]****	1.02 [1.01,1.02]****	1.02 [1.01,1.02]****	-	-
Vaccination	1 vaccine	0.56 [0.24,1.35]	0.44 [0.38,0.52]****	0.43 [0.33,0.56]****	-	-
	2 vaccines	0.31 [0.14,0.68]**	0.43 [0.38,0.50]****	0.35 [0.28,0.44]****	-	-
LFIA result	LFIA negative	1.20 [0.59,2.45]	0.94 [0.83,1.06]	0.89 [0.73,1.10]	-	-
LFIA*Vaccination interaction	LFIA negative : 1 vaccine	0.95 [0.35,2.56]	1.19 [0.98,1.43]	1.23 [0.90,1.69]	-	-
	LFIA negative : 2 vaccines	2.07 [0.82,5.22]	1.19 [0.99,1.42]	1.47 [1.08,2.00]*	-	-

†*Excluded because of insufficient data (N events <8)*

Supplementary Figure 1 Kaplan-Meier plots showing cumulative hazard in LFIA positives (blue line) and LFIA negatives (grey line) stratified by risk group, risk group and age, risk group and vaccination status for A) All-cause mortality, B) Death with underlying COVID-19, C) Death with COVID-19 mentioned anywhere on the death certificate, D) All-cause hospitalisation, E) Emergency hospitalisation, F) Hospitalisation with COVID-19 as the primary diagnosis, G) Hospitalisation with COVID-19 mentioned anywhere on the admission record. 95% pointwise confidence intervals are shown in shaded colour.

A) All-cause mortality

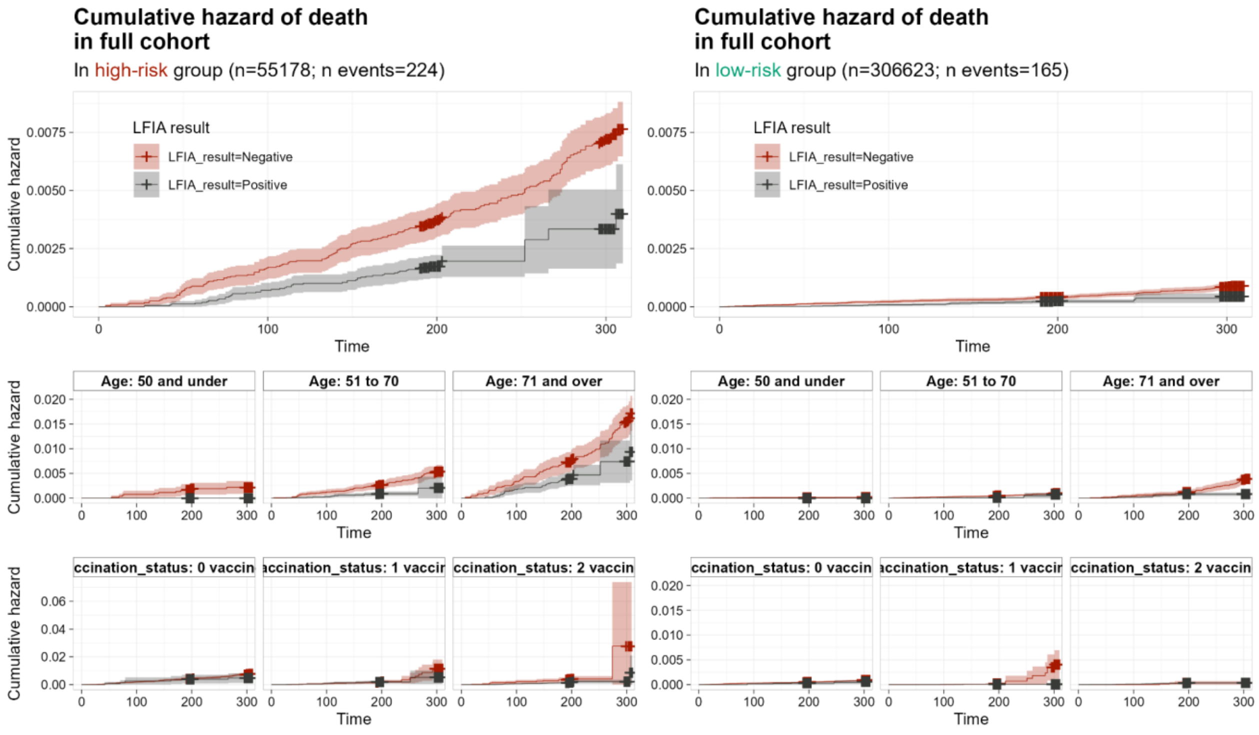

B) Death with underlying COVID-19

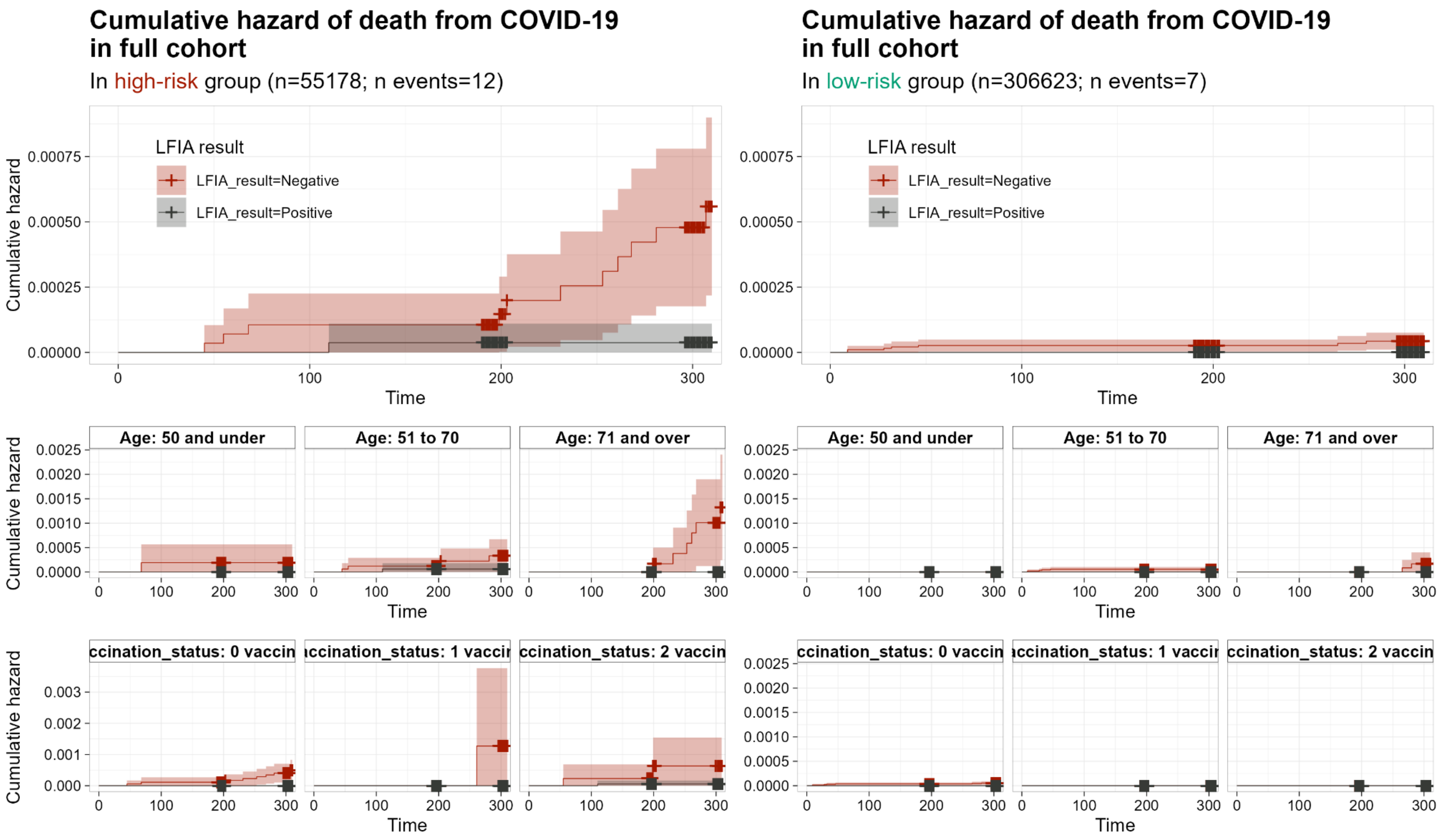

C) Death with COVID-19 mentioned anywhere on the death certificate

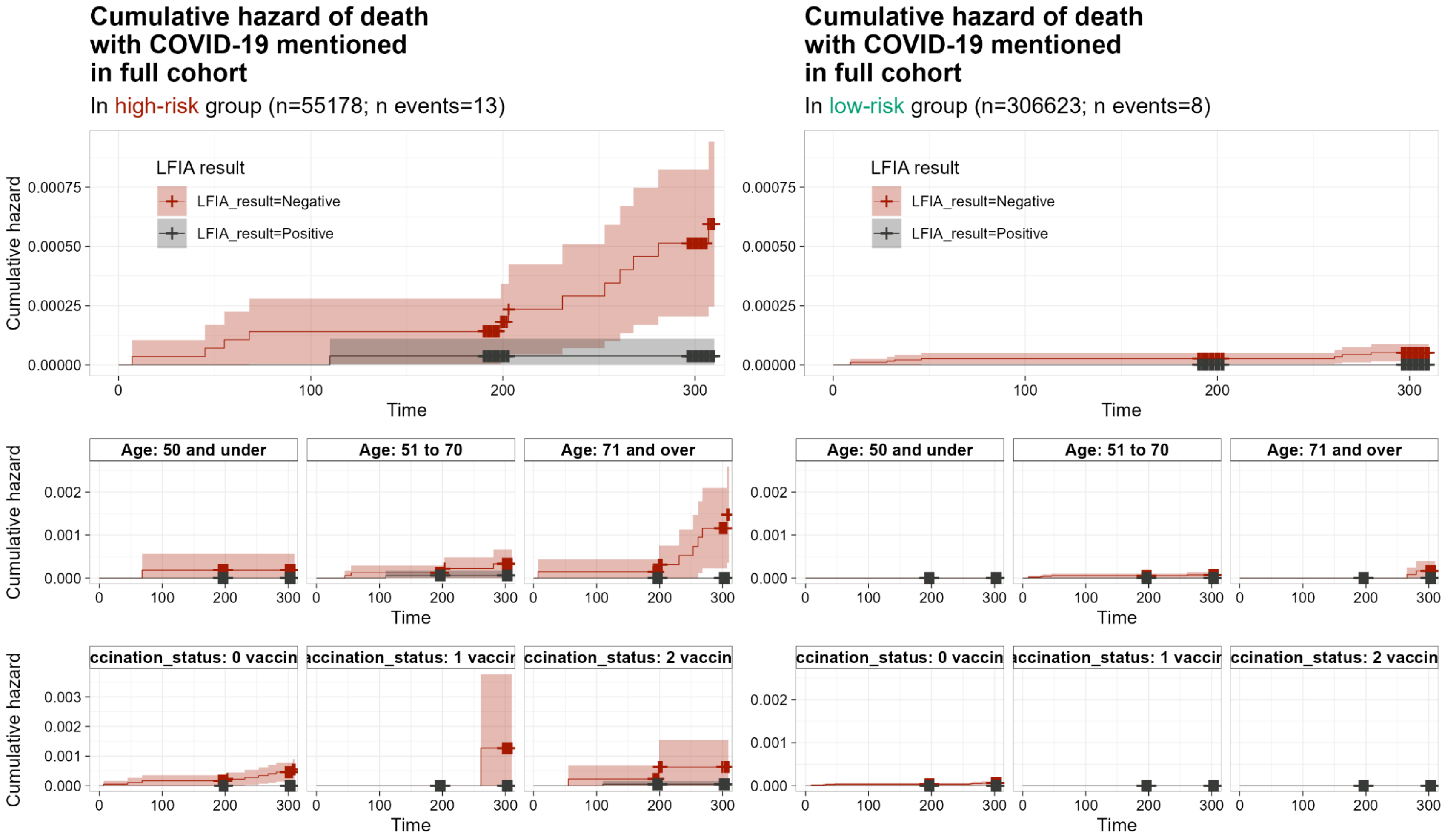

D) All-cause hospitalisation

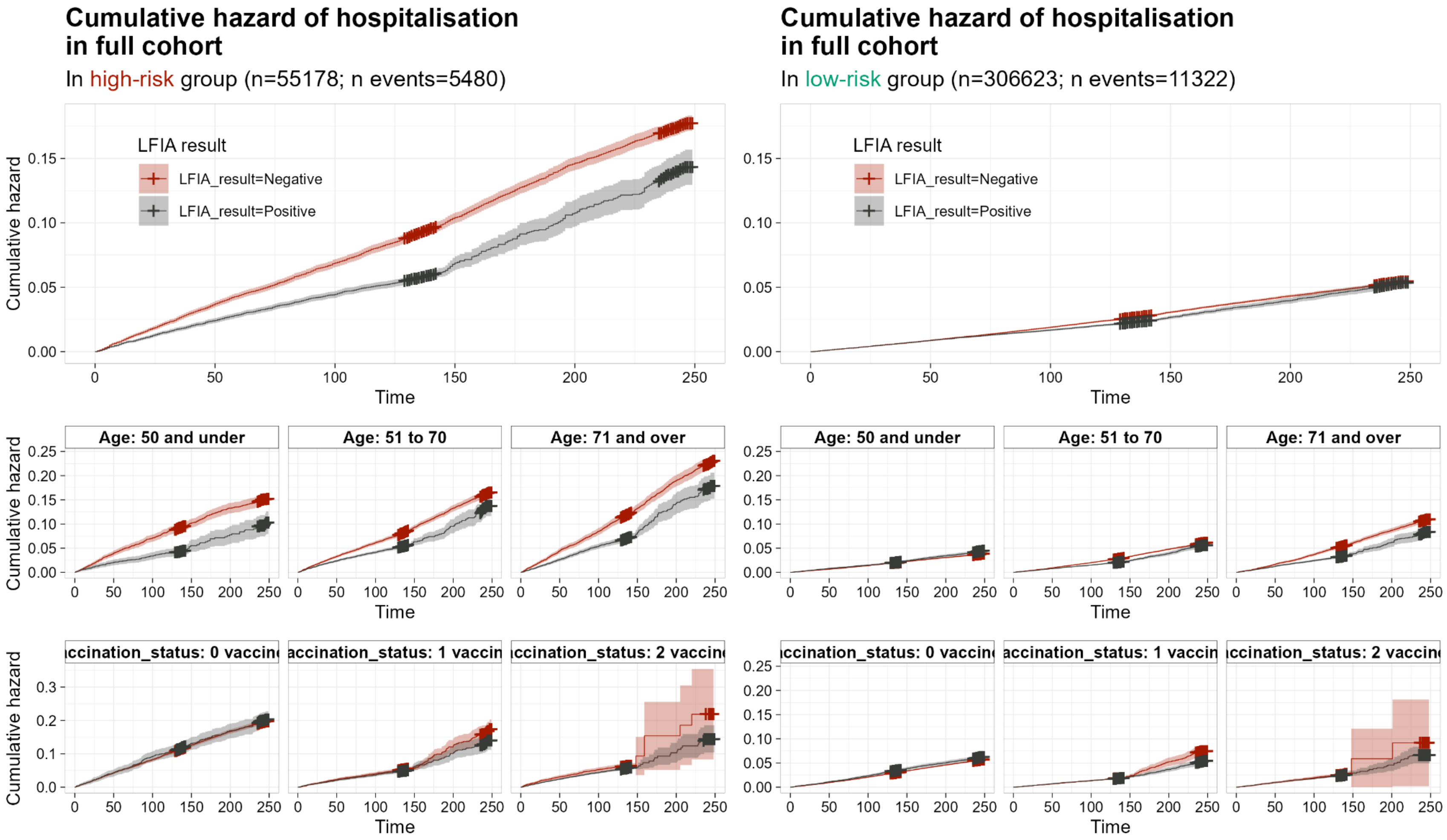

E) Emergency hospitalisation

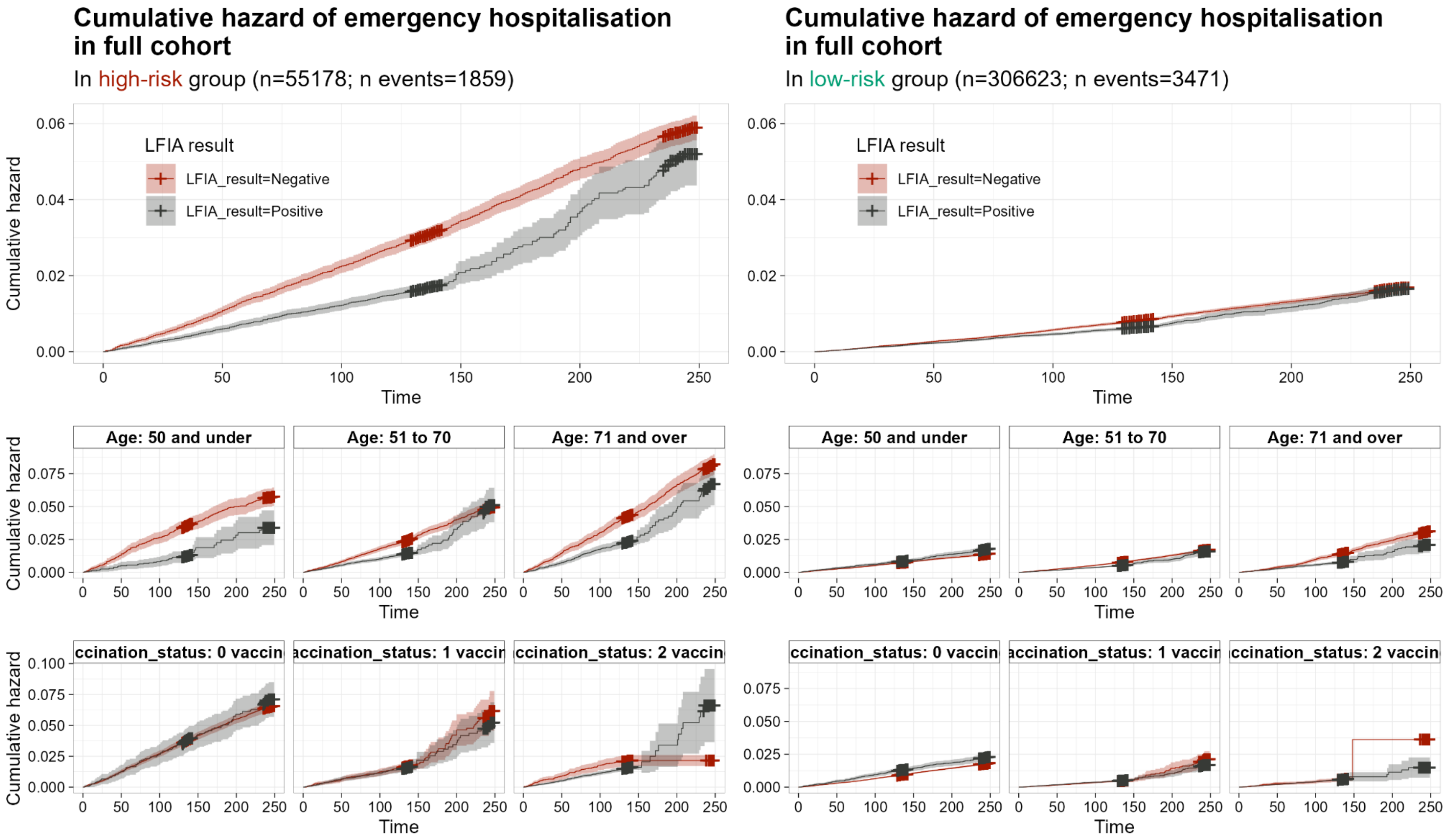

F) Hospitalisation with COVID-19 as the primary diagnosis

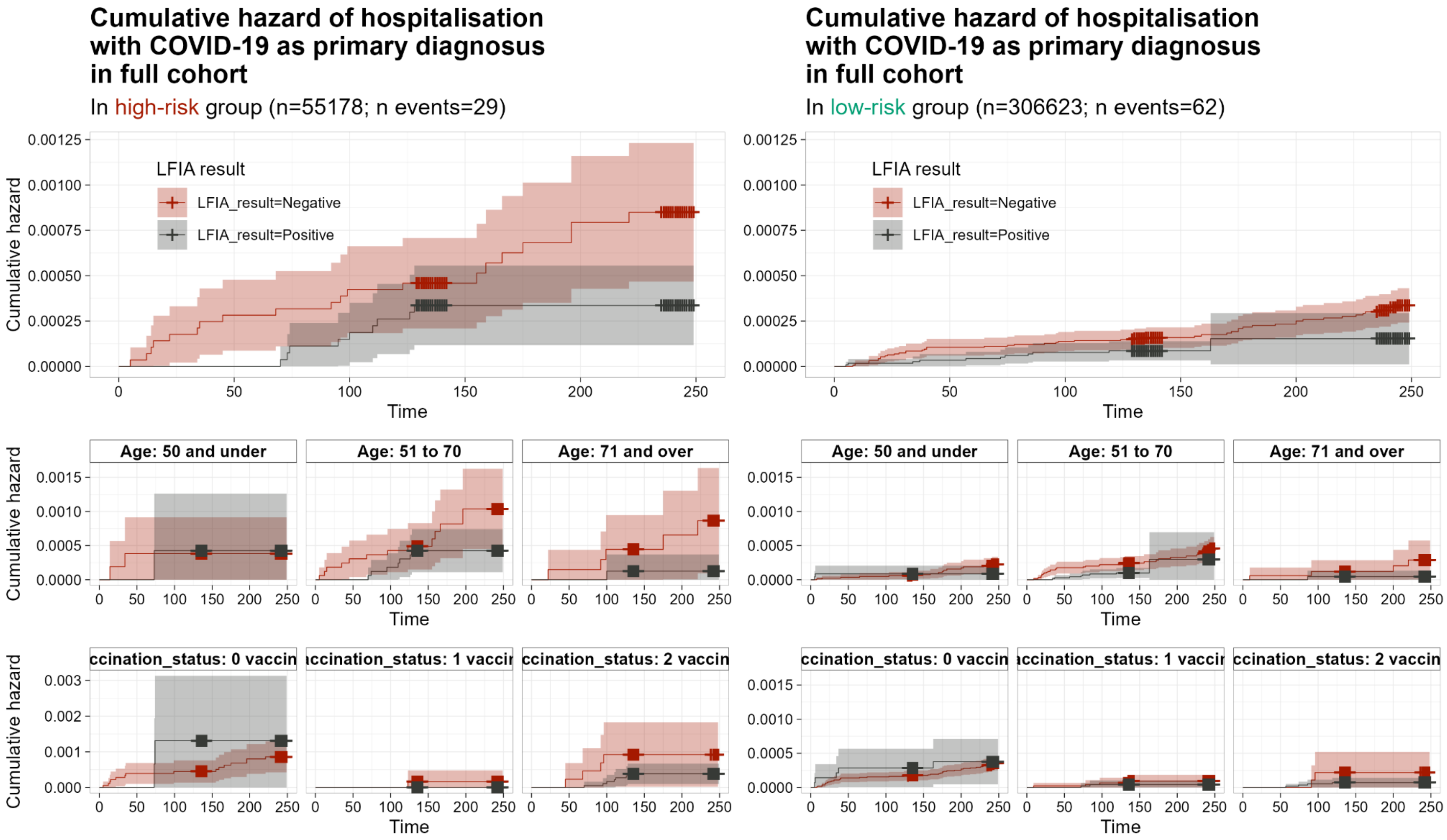

G) Hospitalisation with COVID-19 mentioned anywhere on the admission record

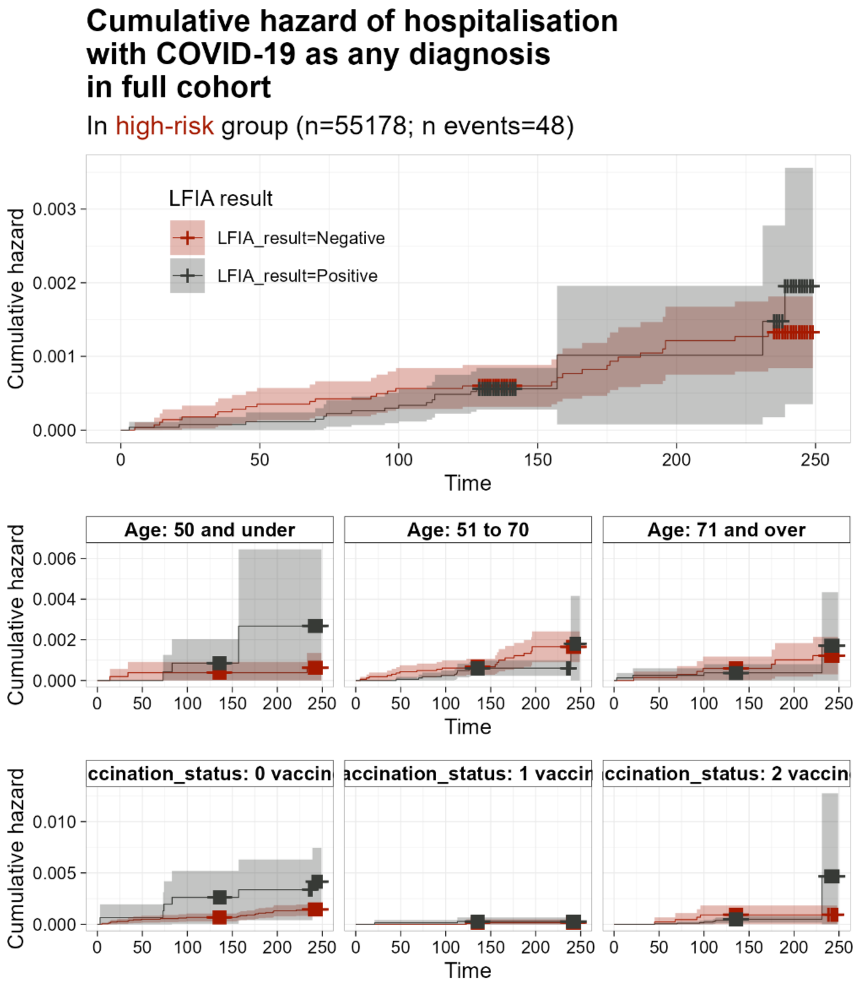

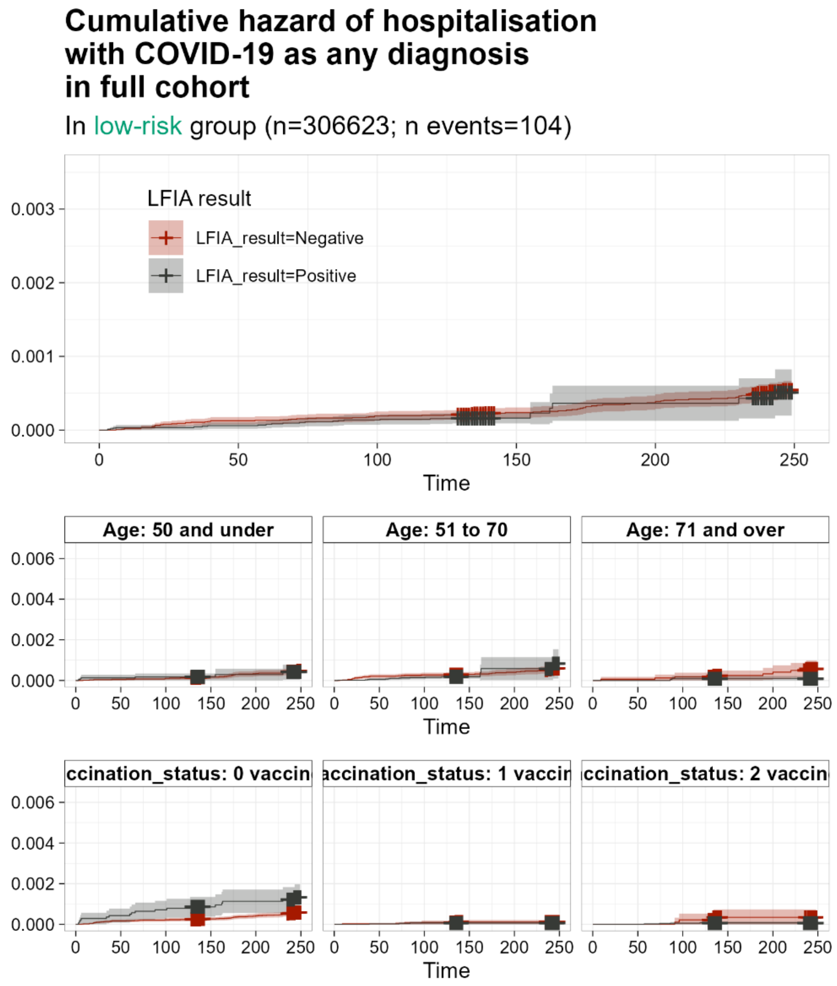

Supplementary Figure 2 Results of penalised Cox modelling with stability selection. Results shown for five main outcomes (l-r: hospitalisation, emergency hospitalisation, hospitalisation with COVID-19, hospitalisation with COVID-19 mentioned, death) and for vaccinated (one or more) cohort (top), high-risk vaccinated cohort (middle) and low-risk vaccinated cohort (bottom).

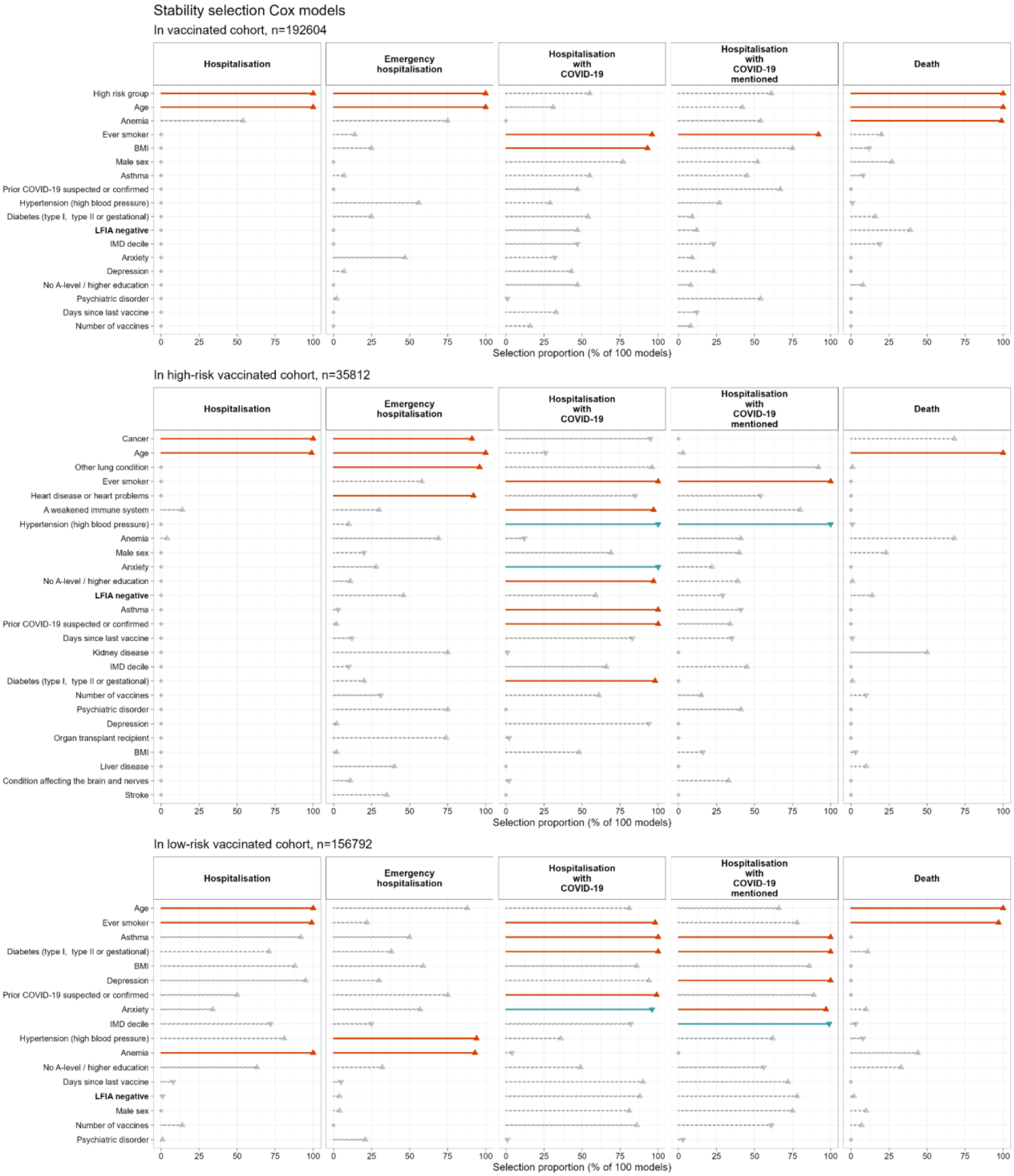

Supplementary Figure 3 ROC curves quantifying the additional predictive power conferred by adding LFIA test result to other survey data from REACT-2 (age, sex, BMI, smoking status, education, deprivation, vaccination status, prior COVID-19 infection, comorbidities). Models built using boosted tree models (Catboost) with 5-fold cross-validation. Outcome is a binary event within follow-up y/n variable. 95% confidence intervals, derived from 1000x bootstrap resamples, are shown in shaded colour around the curves.

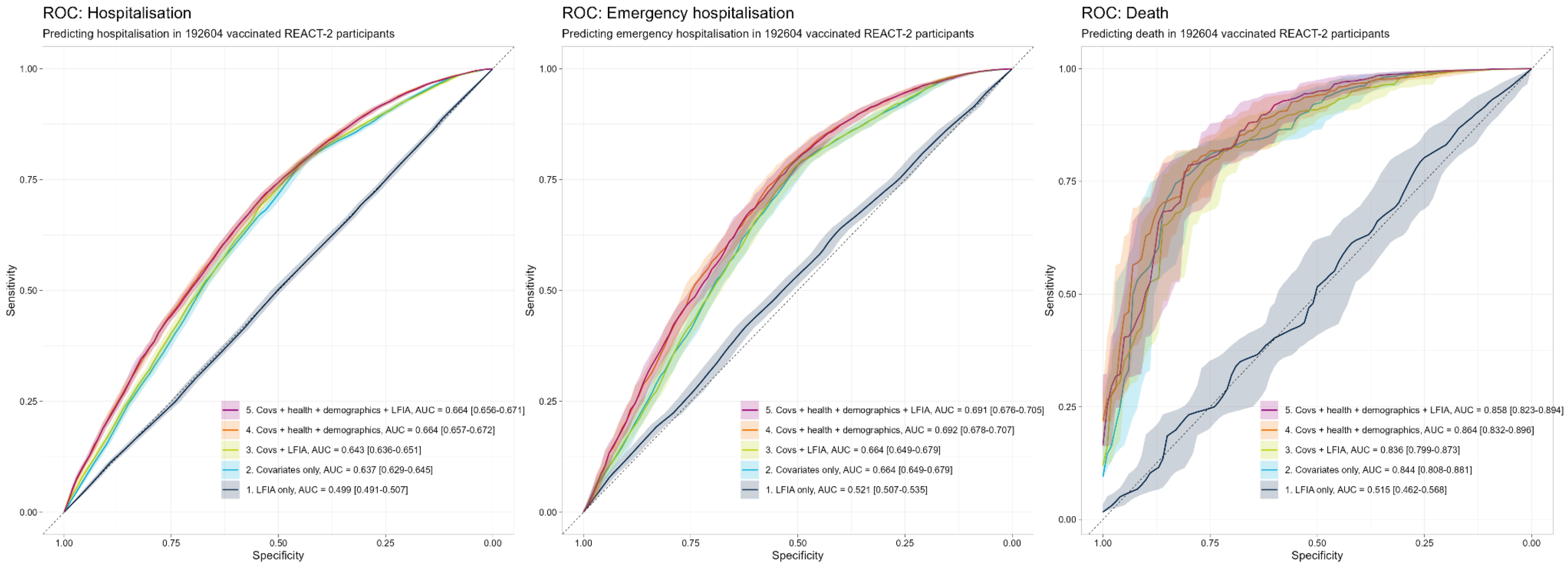

Supplementary methods

S.1 Designation of risk group status

We used our data to replicate, as closely as possible, the criteria for designation of ‘clinically extremely vulnerable’ (CEV) used by the JCVI.[22,23] Participants were assigned to the high-risk group either if they self reported that they had been told that they were CEV by a medical professional, or if they self reported one or more of these health conditions:

- Recipient of organ transplant
- Heart disease or heart problems
- Stroke
- Kidney disease
- Liver disease
- Lung condition such as COPD, emphysema, bronchitis
- Cancer
- Neurological condition
- Weakened immune system / ability to deal with infections

S.2 Description of health outcomes in linked administrative health data

S.2.1 Summary of International Classification of Disease (ICD) 10th edition codes used to identify health outcomes of interest

1. **COVID-19**
   a. U07 (the 4-digit codes present in the dataset were U07.1; U07.2 and U07.4)
2. **Pregnancy**
   a. Pregnancy loss: ICD-10 O00-O08
   b. Delivery/childbirth: ICD-10 O80-O84
3. **Injuries, Accidents and other External Causes**
   a. Injuries: S00-S99; T00-T14
   b. Accidents: V01-V99; W00-W99; X00-X59
   c. Other external causes: T15-T98; X60-X99; Y00-Y98

S.2.2 NHS Digital Hospital Episode Statistics Admitted Patient Care dataset

We obtained data on hospital admissions for the cohort from NHS Digital using the Hospital Episode Statistics (HES) Admitted Patient Care (APC) dataset. HES is the national data warehouse containing information on every admission to an NHS hospital in England (including private patients). The dataset available for research in June 2021, covered 97.11% of the population who were registered with a primary care provider[1]. We excluded admissions where the main diagnosis (in field "diag_03_01") at admission (record with "epiorder" = 1) was for a pregnancy loss or delivery (see S.2.1). We also excluded admissions where the main or secondary diagnosis (in field "diag_03_01" or "diag_03_02") at admission was for an injury, accident or other external cause. (Codes from ICD-10 Chapters V, W, X and Y are not present in the main diagnosis field in this dataset). The remaining dataset contained "hospital admissions for any reason". We then limited the dataset to "unplanned/emergency hospital admissions for any reason" using the method of admission (defined as "Adminmeth" = 21–24, 2A, 2B, 2C, 2D, 28). Hospital admissions with COVID-19 as the main diagnosis were identified from the sub-set of "hospital admissions for any reason" (see S.2.1). Of note, in this dataset the ICD-10 codes

[1] National Data Opt-out, June 2021 - NDRS (digital.nhs.uk)

within the U07 3-digit code were U07.1, U07.2 and U07.4. We used all the available diagnosis code fields (1-20) to identify hospital admissions where COVID-19 was mentioned.

S.2.3 Civil Registrations of Deaths

We obtained records of participant deaths (before December 1, 2021) from NHS Digital using the Civil Registrations of Deaths data that is collected by the UK Office for National Statistics. We analysed all deaths, deaths with COVID-19 recorded as the underlying cause of death and deaths with COVD-19 mentioned anywhere on the death certificate.
