# Supplementary material for: SARS-CoV-2 rapid antibody test results and subsequent risk of hospitalisation and death in 361,801 people
Source: Nat Commun. 2023 Aug 16;14:4957. doi: 10.1038/s41467-023-40643-w (PMC10432566; doi:10.1038/s41467-023-40643-w)
Supplement: Supplementary file 3 — Reporting Summary [file 41467_2023_40643_MOESM3_ESM.pdf]

nature portfolio

Corresponding author(s): Prof Paul Elliott

Last updated by author(s): Jul 28, 2023

Reporting Summary

Nature Portfolio wishes to improve the reproducibility of the work that we publish. This form provides structure for consistency and transparency in reporting. For further information on Nature Portfolio policies, see our Editorial Policies and the Editorial Policy Checklist.

Statistics

For all statistical analyses, confirm that the following items are present in the figure legend, table legend, main text, or Methods section.

n/a	Confirmed	
☐	☒	The exact sample size (*n*) for each experimental group/condition, given as a discrete number and unit of measurement
☐	☒	A statement on whether measurements were taken from distinct samples or whether the same sample was measured repeatedly
☐	☒	The statistical test(s) used AND whether they are one- or two-sided<br>*Only common tests should be described solely by name; describe more complex techniques in the Methods section.*
☐	☒	A description of all covariates tested
☒	☐	A description of any assumptions or corrections, such as tests of normality and adjustment for multiple comparisons
☐	☒	A full description of the statistical parameters including central tendency (e.g. means) or other basic estimates (e.g. regression coefficient) AND variation (e.g. standard deviation) or associated estimates of uncertainty (e.g. confidence intervals)
☐	☒	For null hypothesis testing, the test statistic (e.g. *F*, *t*, *r*) with confidence intervals, effect sizes, degrees of freedom and *P* value noted<br>*Give P values as exact values whenever suitable.*
☒	☐	For Bayesian analysis, information on the choice of priors and Markov chain Monte Carlo settings
☒	☐	For hierarchical and complex designs, identification of the appropriate level for tests and full reporting of outcomes
☒	☐	Estimates of effect sizes (e.g. Cohen's *d*, Pearson's *r*), indicating how they were calculated

Our web collection on statistics for biologists contains articles on many of the points above.

Software and code

Policy information about availability of computer code

Data collection	All data collection for the REACT2 study is captured with Questback (Spring 2020 installation).
Data analysis	All data were analysed using R v4.0.5 (2021). LASSO-penalised Cox regression models was conducted with SHARP R package. Gradient boosted tree modelling was conducted with Catboost, version 1.0.1, (2021).

For manuscripts utilizing custom algorithms or software that are central to the research but not yet described in published literature, software must be made available to editors and reviewers. We strongly encourage code deposition in a community repository (e.g. GitHub). See the Nature Portfolio guidelines for submitting code & software for further information.

Data

Policy information about availability of data

All manuscripts must include a data availability statement. This statement should provide the following information, where applicable:

- Accession codes, unique identifiers, or web links for publicly available datasets
- A description of any restrictions on data availability
- For clinical datasets or third party data, please ensure that the statement adheres to our policy

The datasets generated or analysed, or both, during the current study are not publicly available because of governance restrictions and the identifiable nature of the data. Requests for access to raw data from the REACT study should be addressed to the corresponding authors and will be answered within 12 weeks. The

linked administrative health data that support the findings of this study were provided by NHS Digital under licence for the current study and cannot be made available by the study team.

Research involving human participants, their data, or biological material

Policy information about studies with human participants or human data. See also policy information about sex, gender (identity/presentation), and sexual orientation and race, ethnicity and racism.

Reporting on sex and gender	Data on participant sex was obtained from their NHS registration held by NHS England (the list of people registered with a General Practitioner). Analysis is presented by male/female categories.
Reporting on race, ethnicity, or other socially relevant groupings	Self-reported ethnicity was collected from participants. The coding of the ethnicity variable is described and it is used as a other socially relevant covariate in the analysis.
Population characteristics	Detailed breakdown of the characteristics of the study population are provided in the results tables (table 1) and supplementary files (Supp. Tables 1–3)
Recruitment	REACT-2 is a series of six large representative random samples of the adult population of England between July 2020 and May 2021. Every 6-8 weeks, we obtained a random population sample of adults in England using the National Health Service (NHS) patient list. Personalised invitations were sent to adults aged 18 years and above on the NHS register, to achieve approximately equal numbers of responses from each of the 315 lower-tier local authority areas in England. Registration was done by telephone or on line, and recruitment ceased when ~120,000 people had signed up. In the sixth and final round (May 2021), a boosted sample of adults aged 50 and above was obtained, with 749,225 invitations sent out resulting in 255,750 (34.1%) registrations. There is a risk of participation bias from this type of sampling strategy.
Ethics oversight	Ethical approval for the study was obtained from South-Central Berkshire B Research Ethics Committee reference numbers 20/SC/0206; 21/SC/0163).

Note that full information on the approval of the study protocol must also be provided in the manuscript.

Field-specific reporting

Please select the one below that is the best fit for your research. If you are not sure, read the appropriate sections before making your selection.

☒ Life sciences ☐ Behavioural & social sciences ☐ Ecological, evolutionary & environmental sciences

For a reference copy of the document with all sections, see nature.com/documents/nr-reporting-summary-flat.pdf

Life sciences study design

All studies must disclose on these points even when the disclosure is negative.

Sample size	Sample size was 361,801 participants from REACT-2 rounds 5 (January - February 2021) and 6 (May 2021) who had a valid LFIA result (positive or negative) and provided consent for linkage to their administrative health data. The REACT-2 study is powered conservatively to give information on every LTLA in England (n=315), in each round, under the assumption that prevalence in each Local Authority is independent. Full information on sample size calculation and rationale is available in the protocol paper: https://wellcomeopenresearch.org/articles/5-200/v2.
Data exclusions	Participants reporting 3 or more vaccine doses at enrolment were excluded. Analyses of risk hospitalisation and mortality were restricted to (N=192,637) participants with one or more vaccine doses at least 14 days prior to LFIA (ie excluding 169,164 participants)
Replication	Replication analysis was not directly undertaken, although multivariable model performance was evaluated on a holdout data set.
Randomization	Randomisation not applicable to a cross-sectional study design.
Blinding	As this was a cross-sectional study design with no grouping, no blinding was or could be applied to the researchers.

Reporting for specific materials, systems and methods

We require information from authors about some types of materials, experimental systems and methods used in many studies. Here, indicate whether each material, system or method listed is relevant to your study. If you are not sure if a list item applies to your research, read the appropriate section before selecting a response.

Materials & experimental systems

n/a	Involved in the study	
☒	☐	Antibodies
☒	☐	Eukaryotic cell lines
☒	☐	Palaeontology and archaeology
☒	☐	Animals and other organisms
☒	☐	Clinical data
☒	☐	Dual use research of concern
☒	☐	Plants

Methods

n/a	Involved in the study	
☒	☐	ChIP-seq
☒	☐	Flow cytometry
☒	☐	MRI-based neuroimaging
